# Large-scale MHC class II genotyping of a wild lemur population by next generation sequencing

**DOI:** 10.1007/s00251-012-0649-6

**Published:** 2012-09-05

**Authors:** Elise Huchard, Christina Albrecht, Susanne Schliehe-Diecks, Alice Baniel, Christian Roos, Peter M. Kappeler Peter, Markus Brameier

**Affiliations:** 1 Behavioral Ecology and Sociobiology Unit, German Primate Center, Kellnerweg 4, Göttingen, Germany; 2 Courant Research Center Evolution of Social Behavior, University of Göttingen, Kellnerweg 6, Göttingen, Germany; 3 Primate Genetics Laboratory, German Primate Center, Kellnerweg 4, Göttingen, Germany; 4 Gene Bank of Primates, German Primate Center, Kellnerweg 4, Göttingen, Germany; 5 Department of Zoology, University of Cambridge, Downing Street, CB2 3EJ Cambridge, UK

**Keywords:** MHC, Genetic diversity, Next generation (454) sequencing, *Microcebus murinus*, Positive selection

## Abstract

The critical role of major histocompatibility complex (MHC) genes in disease resistance, along with their putative function in sexual selection, reproduction and chemical ecology, make them an important genetic system in evolutionary ecology. Studying selective pressures acting on MHC genes in the wild nevertheless requires population-wide genotyping, which has long been challenging because of their extensive polymorphism. Here, we report on large-scale genotyping of the MHC class II loci of the grey mouse lemur (*Microcebus murinus*) from a wild population in western Madagascar. The second exons from MHC-DRB and -DQB of 772 and 672 individuals were sequenced, respectively, using a 454 sequencing platform, generating more than 800,000 reads. Sequence analysis, through a stepwise variant validation procedure, allowed reliable typing of more than 600 individuals. The quality of our genotyping was evaluated through three independent methods, namely genotyping the same individuals by both cloning and 454 sequencing, running duplicates, and comparing parent–offspring dyads; each displaying very high accuracy. A total of 61 (including 20 new) and 60 (including 53 new) alleles were detected at DRB and DQB genes, respectively. Both loci were non-duplicated, in tight linkage disequilibrium and in Hardy–Weinberg equilibrium, despite the fact that sequence analysis revealed clear evidence of historical selection. Our results highlight the potential of 454 sequencing technology in attempts to investigate patterns of selection shaping MHC variation in contemporary populations. The power of this approach will nevertheless be conditional upon strict quality control of the genotyping data.

## Introduction

Major histocompatibility complex (MHC) molecules are cell surface glycoproteins that play a critical role in the vertebrate immune system by binding “self” and “non-self” antigenic peptides and presenting them to T-lymphocytes, which then initiate an immune response against pathogens. MHC genes are the most polymorphic loci of the vertebrate genome, and high levels of variation characterize the extent of sequence variation among alleles as well as the number of genes within and across species (Hughes and Yeager 1998; Kappeler and Fichtel 2012). Because nonsynonymous substitutions are typically concentrated in the antigen-binding sites (ABS) of the MHC molecules, the extent of allelic polymorphism is thought to reflect parasite-driven selection (Hughes and Hughes 1995; Hughes and Nei 1988). Individual MHC molecules can only bind a subset of antigens, and gene duplications and polymorphism at the ABS are thus thought to reflect adaptations enabling individuals to respond to a greater variety of antigens. In addition, MHC genes have been found to be involved in various non-immune functions, including the regulation of materno–foetal interactions (Ober 1998, 1999; Ober et al. 1993), a potential role in fertilization (Ziegler et al. 2005) and the expression of body odours (Kwak et al. 2011), which might all influence mating behaviour and reproductive outcome (Alberts and Ober 1993; Ziegler et al. 2010). As such, it is likely that sexual selection plays a significant role, alongside natural selection, in maintaining MHC polymorphism. MHC has therefore attracted interest from biologists across various fields, including immunology and genetics, but also reproductive and evolutionary biology as well as conservation biology.

The mammalian MHC represents a large gene cluster typically divided into three main regions: class I, II, and III (Kelley et al. 2005). Class I genes are expressed on nearly all nucleated cells and act in defence against intracellular (mainly viral) pathogens, whereas class II genes are expressed on immune cells and involved in detecting (mainly bacterial and parasitic) antigens from the extracellular environment. Class III genes are mainly involved in coding secreted protein from the innate immune system, such as components of the complement or cytokines. The MHC class II genes of many non-human primates were investigated extensively in the past two decades (e.g., across species: Bontrop et al. 1999; Garamszegi et al. 2009; Gyllensten et al. 1991; Klein et al. 1993; Otting et al. 2002) (in various Old World monkeys: de Groot et al. 2004; Doxiadis et al. 2000; Huchard et al. 2008; Lukas et al. 2004; Otting et al. 2002; Slierendregt et al. 1992, 1994). Although species differ in the number and organisation of loci, in the number of alleles (‘allelic diversity’) and of divergence among these alleles (‘sequence diversity’), long-term retention of allelic lineages within the primate order reflects shared evolutionary history combined with vulnerability to similar pathogen pressures (Bontrop 2006).

MHC genes have been less intensively studied in the Malagasy primate radiation (Lemuriformes) than in other primates. Nevertheless, integrating lemurs into comparative patterns of MHC evolution is interesting due to their long isolated evolutionary history (Go et al. 2002, 2003; Perelman et al. 2011). The grey mouse lemur (*Microcebus murinus*), a small nocturnal and solitary forager, is the best studied lemur species concerning MHC diversity (Averdam et al. 2011; Schad et al. 2004, 2005; Schwensow et al. 2010). It represents an interesting model for the study of MHC polymorphisms for several reasons. First, mouse lemurs are regarded as a promising biomedical model, because they are easily bred in captivity, and are taxonomically and physiologically closer to humans than rodents. Second, studies in wild populations revealed that the MHC class II region is extremely polymorphic (Schad et al. 2004; Schwensow et al. 2008), and influences nematode resistance (Schad et al. 2005; Schwensow et al. 2010) as well as reproductive strategies (Schwensow et al. 2008). Finally, a recent genomic analysis of MHC class II organisation confirmed that DRB and DQB loci are the most variable parts of the class II region, and that, in contrast to most primates for which the organisation of the class II region is known, these two loci are non-duplicated (Averdam et al. 2011). This fact may facilitate the use of population genetic tools to examine the evolutionary drivers of MHC polymorphism, which is rarely possible in non-model organisms with duplicated loci, as it is impossible to assign sequences to loci (Bernatchez and Landry 2003).

Species harbouring single-locus MHC systems are also interesting to evaluate the quality of MHC-genotyping methods. Evolutionary studies of the MHC in non-model organisms have long been plagued by technical challenges associated with the need for high-resolution, accurate and large-scale genotyping of a complex multilocus system (Babik 2010). Because it typically requires simultaneous analysis of multiple co-amplifying loci, accurate separation of closely related sequences, which can often diverge by point mutations, has traditionally been achieved through cloning and sequencing. Although this has long represented the “gold standard” for defining MHC polymorphisms in non-model organisms, it is costly and time-consuming, and thus very impractical for large sample sizes (Babik 2010). The recent introduction of Next Generation Sequencing (NGS) to MHC genotyping, in contrast, shows promise to circumvent these problems. Indeed, the use of tagged polymerase chain reaction (PCR) primers allows the identification of individual amplicons, which can be sequenced in parallel (Babik et al. 2009). However, the utility of NGS technology comes at a cost, namely, the frequent occurrence of genotyping errors. Pioneering studies on an increasing range of organisms have suggested that this difficulty might be overcome by a stringent quality control, allowing sorting true and false alleles (Babik et al. 2009; Galan et al. 2010; Sepil et al. 2012; Wegner 2009; Zagalska-Neubauer et al. 2010). Yet, it remains difficult to evaluate the reliability of a protocol using an organism for which MHC organisation is unknown, because (1) the number of distinct sequences expected per individual is unknown, and (2) the sequences cannot be assigned to particular loci. It might thus prove difficult to detect the presence of null alleles or the presence of artefactual alleles (AA) remaining after quality screening. As such, using a species possessing a single-locus can shed light on the reliability and accuracy of MHC genotyping using NGS technology.

Here, we describe an approach for large-scale and cost-effective genotyping of MHC class II DRB and DQB loci in natural mouse lemur populations. We used 454 pyrosequencing to genotype both loci simultaneously in several hundred mouse lemurs, and applied a stepwise variant validation procedure to separate true alleles from sequencing artefacts. We evaluated the quality of our genotyping through (1) comparing results obtained with this approach to those obtained through cloning and sequencing for the same individuals, (2) sequencing duplicates, and (3) estimating mismatch rates for several hundreds of parent–offspring triads. We also compared the strength of selection at DRB and DQB loci through tests of historical selection. Finally, we investigated the extent to which our sampling scheme allowed estimating the level of MHC diversity (i.e., allelic richness) present in this population.

## Methods

### Samples and DNA extraction

For MHC genotyping, genomic DNA was extracted from tissue samples from 665 wild grey mouse lemur (*M. murinus*) individuals from a population living between 2000 and 2010 in Kirindy Forest, located about 60 km northeast of Morondava in western Madagascar (Kappeler and Fichtel 2012). Members of this population have been regularly captured and marked individually with sub-dermal transponders within a 9-ha study area since 1994, referred to below as the central study area, and within an additional 21 ha surrounding the central study area since 1999 (Eberle and Kappeler 2002, 2004a, b). DNA was isolated from ear biopsies following standard protocols (Qiagen QIAmp DNA Mini Kit no. 51306). Parentage relationships (either mother or father) have been established using microsatellite markers for 1094 individuals of this population, using 13 loci with an average of 21 alleles per locus (standard deviation [SD] = 9.48, range = 13–43). Both paternal and maternal identities have been identified for 333 offspring (unpublished data). Methods used to genotype individuals and to establish maternities and paternities have been described in detail elsewhere (Eberle and Kappeler 2004b).

### Cloning and sequencing

The complete set of MHC-DRB sequences were characterised for five individuals, including a mother–offspring dyad. A PCR was performed on individual genomic DNA with the primers JS1 and JS2, amplifying the most variable part of the second exon of the MHC-DRB gene (Schad et al. 2004). Overall, 30 to 40 ng of genomic DNA in 50 μl of Buffer E from an Optimization Buffer kit (Bioline, Luckenwalde, Germany), 25 pmol of each primer and 1 unit of Bio-X-Act short DNA polymerase (Bioline) were used for amplification. PCR conditions consisted of 35 cycles of 90 s denaturation at 94 °C, 90 s annealing at 54 °C, and 90 s extension at 74 °C. Two microliters of the 182 bp PCR product was purified on a 1.5 % agarose gel before cloning, using a Topo TA Cloning kit (Invitrogen, Darmstadt, Germany) following the manufacturer’s instructions. For each individual, at least 30 bacterial colonies were purified with a Qiagen plasmid preparation kit and sequencing was directly performed on an ABI 310 DNA sequencer using the Big Dye Terminator kit and the primers M13R-pUC (5′-CAGGAAACAGCTATGAC-3′) and M13F (5′-GTAAAACGACGGCCAGT-3′).

### 454 sequencing

Amplification of DQB used the primers Mimu-DQB-nested-fwd (CTCTGCTACTTCACYAACGG) and Mimu-DQB-nested-rev (TTGTGTCTGCACACCGTGT) (Averdam et al. 2011). These primers are located at the beginning and end of the second exon of the DQB gene and amplify its most variable part (Averdam et al. 2011). In addition to these template-specific primers, the 454 sequencing system requires the addition of a 25-mer 5′-portion whose sequence is designed according to manufacturer’s instructions (Roche Applied Science, Mannheim, Germany) for binding to the DNA Capture Beads and for annealing the emPCR Amplification Primers and the Sequencing Primer; in addition, this 5′-part must end with the sequencing key “TCAG” used for amplicon sequencing. Two kinds of such primers allow for the directional sequencing of the target sequence from either end. Finally, a 10-bp tag identifying an individual can be added between the sequencing key and the template-specific sequence. In total, the length of the amplified fragment including primers was 286 bp for DRB and 276 bp for DQB, with primer length ranging from 54 to 59 bp. To genotype 96 individuals, we thus used ten different individual tags in the forward primer, combined with ten different tags in the reverse primers. Identification by unordered tag pairs allowed us to minimize the number (and associated costs) of individual tags ordered (20 per locus, rather than 96). We used 20 out of 10^8^ possible tags, which leaves a minimal probability (<10^−8^) for a sequence to be assigned to the wrong individual due to a typing mistake in the individual tag (according to manufacturer’s instructions).

PCR was performed as described above and 2 μl of a subset of the PCR products was electrophoresed on a 1 % agarose gel after PCR to confirm successful amplification of DNA samples. PCR products were purified using Agencourt AMPure XP (Beckman Coulter Genomics, Bernried, Germany) and their concentrations measured by fluorometry using the Quant-iT PicoGreen dsDNA Assay Kit (Invitrogen) and adjusted accordingly. Equimolar amounts of 96 individual amplicons were pooled for a given locus (i.e., DRB or DQB). Here and later, we define as ‘amplicon’ the pool of reads amplified from a same individual during a same PCR. DRB and DQB libraries were then pooled in equal amount and sequenced together in both directions on a Roche® GS Junior System. Due to an unequal distribution of sequences between DRB and DQB loci in these sequencing results, with a large excess of DQB (over DRB) sequences, we re-adjusted the pooling strategy for the next runs, by pooling 1 equivalent of the DQB amplicon library with two equivalents of the DRB amplicon library. We conducted seven successive sequencing runs in total. We increased the size of the DRB library for run numbers 6–7 by ordering an extra two forward and reverse tags, thus adding an extra 48 amplicons in each. A total of 768 DRB amplicons, including 76 duplicates and seven triplicates, and 672 DQB amplicons, were included in the sequencing process.

### 454 library pre-processing

The various steps involved in the 454 library pre-processing and in the allele filtering are summarised in Table 1. After the initial quality assessment using standard settings of the 454 software, the following procedure was used to increase stringency of the quality control. First, only sequences showing perfect match to both target-specific (forward and reverse) primers and fully containing both (forward and reverse) tags were extracted from the raw read library, i.e., the multi-FASTA file produced as the result of a 454 run. After cutting off primers and tags, the library file was compressed by removing identical reads found in the same individual. For further processing, the number of read copies was noted in the FASTA comment of each sequence, together with the individual identifier (derived from the tag pair). Note that only perfectly identical reads (without mismatches) were merged. In an initial filtering step we removed all sequences that occurred less than five times in one individual. All pre-processing steps were carried out by custom Perl scripts.

**Table 1:** Summary table of the stepwise variant validation procedure. Every step was strictly similar among both loci

Stage	Step no.	Filter applied	*N*. reads	*N*. amplicons	*N*. individuals	*N*. variants
Library pre-processing	1	Quality assessment of the 454 software	860,000	_	_	_
	2	Elimination of sequences without full-length (forward and reverse) tags	780,000	_	_	_
	3	Elimination of sequences without perfect match to both target-specific (forward and reverse) primers	700,000	_	_	_
	4	Cut-off primers	700,000	_	_	_
	5	Elimination of sequences with less than 5 reads within individual amplicons	558,000	726 (DRB) 646 (DQB)	659 (DRB) 646 (DQB)	_
	6	Elimination of individuals with less than 18 reads	558,000	711 (DRB) 643 (DQB)	654 (DRB) 643 (DQB)	_
	7	Cutoff DRB and DQB fragments to the same region for alignment (163–169 bp)	558,000	711 (DRB) 643 (DQB)	654 (DRB) 643 (DQB)	706 (DRB) 827 (DQB)
Allele sorting	8	Elimination of variants with MPAF < 5 %	512,000	711 (DRB) 643 (DQB)	654 (DRB) 643 (DQB)	61 (DRB) 60 (DQB)
	9	Elimination of RA to keep only TMCA	512,000	711 (DRB) 643 (DQB)	654 (DRB) 643 (DQB)	61 (DRB) 60 (DQB)

Accurate genotyping requires a minimum number of reads per locus and individual. A probabilistic model was recently proposed to determine the confidence level of genotyping for each individual (Galan et al. 2010). The confidence level (*f*) depends on the values of *r*, *n* and *m*, with *r* being the minimum copy number of each true variant, *n* the total number of sequences, and *m* the maximal number of variants for the gene. We used the program ‘Negative Multinomial’ (Galan et al. 2010) freely available online (http://www.lirmm.fr/~caraux/Bioinformatics/NegativeMultinomial/) to estimate the minimum number of sequences required per individual for reliable genotyping and calculated the number of sequences that are necessary for amplifying all the variants at least three times (*r* = 3) from one locus (*m* = 2), giving a confidence level (*f*) of 0.95. We found that the minimum number of sequences (*n*) required per individual was 18. Amplicons yielding less than 18 reads were thus discarded at this stage.

### Sorting true and false alleles

Based on previous published studies using 454 technology to sequence MHC genes (e.g., Babik et al. 2009; Galan et al. 2010; Wegner 2009; Zagalska-Neubauer et al. 2010), it is likely that a fraction of sequences might still contain errors and could be misidentified as rare true alleles (TA). A procedure allowing identification and exclusion of AA was applied, inspired from protocols proposed by these studies. We initially established the mean per-amplicon frequency (MPAF) of any given allele (the proportion of reads from an amplicon assigned to this allele, averaged across the amplicons possessing this allele), assuming that AAs introduced by genotyping mistakes would occur at low frequency within individual amplicons and thus display low MPAF. Based on this assumption, we took three different approaches to define a criterion allowing us to discriminate TA from AA reliably.

First, we compared the MPAF of the two most common alleles (TMCA) with the remaining alleles (RA) within each individual amplicon, assuming that the RA would mostly contain AA. We thus expected MPAF to be much higher for the TMCA than for the RA, which would allow us to derive an estimate of the threshold MPAF under which alleles are likely to represent AA. Second, for sequences with relatively low MPAF, we compared the nucleotide sequence with those of other, most common alleles (later referred to as “parental sequences”) from the same amplicon, in order to check whether they could represent sequencing artefacts, namely a chimera of two parental sequences or a point-mutation variant of any parental sequence. We randomly selected and examined 30 sequences with MPAF <0.01, and then similarly examined all sequences with 0.03< MPAF <0.05, as well as all sequences with 0.05< MPAF <0.25. In the latter scenario, we expected the sequence of an AA to either deviate from a more common allele by a point mutation, or to represent a chimera of two more common alleles. This would allow us to derive a second estimate of the threshold MPAF under which alleles are likely to represent AA. Finally, we correlated the MPAF with the number of individuals possessing a given allele (allelic frequency), assuming that a genotyping mistake resulting in a same AA would rarely occur independently in many individuals. We expected that because most alleles retrieved from a unique or few amplicon(s) would also have low MPAF, a positive relationship between MPAF and allelic frequency observed before allele sorting would disappear after allele sorting (at least if our sorting procedure is efficient).

Note that our stepwise validation procedure retained alleles that were possessed by only one individual if these displayed relatively high per-amplicon frequency (>0.05) and were not identified as sequencing artefacts through comparison with more common alleles from the same amplicon. By contrast, traditional approaches in MHC typing require amplification from at least two independent PCRs to ensure reliability of a new allele description. This procedure would inevitably lead to the elimination of true alleles, thus introducing a risk of biasing further population genetic analyses based on this dataset through the occurrence of null alleles. Nevertheless, sequences retrieved from only one individual were not submitted to Genbank to preclude publication of unreliable sequences (12 DRB and 13 DQB alleles). Their nucleotide sequence is provided in the Appendix for information.

### Validation of genotyping accuracy

We then examined all individuals that possessed more than two alleles after this control check. We ran duplicates by re-amplifying and re-sequencing these individuals that were found to possess more than two alleles in the first four runs, as well as examined patterns of inheritance by comparing parent–offspring dyads in order to evaluate the possibility that these alleles are TAs arising from a non-fixed duplication of the DRB or DQB locus in the population.

Once the final genotypes were determined for each individual, we validated the quality of our genotyping in three steps. In the first step, we compared the DRB genotypes obtained through 454 sequencing and cloning for five individuals. In the second step, we established the intra-individual repeatability of 454 sequencing by comparing genotypes among duplicates obtained from independent amplifications, so new amplicons, of the same DNA sample. In the last step, we compared the genotypes of mother or father–offspring pairs, and of mother–father–offspring triads.

### Sequence analysis and phylogenetic tree

Pairwise distances among nucleotide and amino-acid sequences were computed in MEGA 5.0 (Koichiro et al. 2011). *Mimu*-DRB and -DQB sequences were compared in a phylogenetic tree constructed by the neighbour-joining algorithm based upon distances corrected with the Jukes–Cantor method using MEGA. Bootstrap analyses using 500 replications were performed to determine the repeatability of the sequence alignment.

### Basic population parameters

Estimation of linkage disequilibrium between DRB and DQB loci was tested using a likelihood ratio test where the likelihood of the sample evaluated under the hypothesis of no association between loci (linkage equilibrium) is compared to the likelihood of the sample when association is allowed (Slatkin and Excoffier 1996). The significance of the procedure is found by computing the null distribution of this ratio under the hypothesis of linkage equilibrium using a permutation procedure (here with 100,000 permutations) implemented in the software ARLEQUIN 3.5.1.3 (Excoffier and Lischer 2010). Deviations from Hardy–Weinberg equilibrium were tested using the exact *U*-score test of Rousset and Raymond (Rousset and Raymond 1995), where the alternative hypothesis is heterozygote excess. This test was carried out including the whole sample at each locus individually.

### Detecting positive selection

Next, we investigated the presence of positively selected sites (PSS), characterised by ω = *d*_N_/*d*_S_ > 1, where *d*_N_ and *d*_S_ are the relative amounts of substitutions at non-silent and silent codon sites, in *Mimu*-DRB and -DQB amino acid sequences. We compared the null model, where *ω* < 1 and varies according to the beta distribution (model M7), and a model allowing an additional class of sites where *ω* > 1, to account for the possible occurrence of PSS (model M8) using a Likelihood Ratio Test (LRT) (Yang et al. 2000). If M8 fits the dataset better than M7, PSS were subsequently identified using an empirical Bayesian method. Analyses were carried out using the software CODEML, implemented in the package PAML 3.14 (Yang 1997). M7 and M8 have proved to be more robust towards intragenic recombination than other implemented models, as well as the Bayes’ prediction of sites under positive selection (Anisimova et al. 2003).

Values of *d*_S_, *d*_N_ and their standard errors were then estimated using a second approach: the evolutionary pathways method (Nei and Gojobori 1986) implemented in MEGA 5.0, applying the correction of Jukes and Cantor (1969) for multiple hits. In counting the pairwise number of silent and non-silent substitutions in a set of sequences, the evolutionary pathways method considers all possible evolutionary pathways (excluding termination codons) leading from one codon to another as equally probable and therefore makes fewer assumptions than other methods. This method is expected to provide conservative (minimum) estimates of numbers of substitutions and, therefore, to be conservative compared to the positive selection hypothesis (*ω* > 1) (Nei and Kumar 2000). Separate tests were conducted for sites predicted to be involved in antigen recognition, assuming concordance to human ABS (Bondinas et al. 2007) as well as for non-ABS sites, and then for PSS and non PSS. A global test was also conducted using all sequences to calculate the overall values of *d*_N_ and *d*_S_.

### Estimation of population allelic richness

Finally, we investigated the extent to which our sampling effort allowed estimating population allelic richness. We conducted simple permutation tests to evaluate the number of alleles detected for a given sampling effort. We initially selected 20 individuals randomly and counted the number of distinct alleles detected. This procedure was repeated 100 times to calculate a mean and SD for each sampling effort. It was then implemented through an incremental process adding 20 individuals at each step until 600. Graphical exploration of the resulting plot, displaying the number of alleles detected for different sampling schemes, provided an indication of the minimal sampling effort required to estimate the population allelic richness through the asymptotic value of the number of alleles detected.

## Results

### Sequencing output

The seven respective sequencing runs yielded a total of around 860,000 reads, including 700,000 with perfectly matching target-specific primers. After the initial filtering (Table 1), there were about 558,000 reads (291,000 DQB and 267,000 DRB) left with minimum five copies in one individual.

During sequencing, a recombination occurring between the end of the DRB fragment (4 nucleotides before the start of the DRB reverse primer) and the reverse DQB specific primer led to chimeric sequences associating the DRB forward primer with the DQB reverse primer, in roughly 155,000 (22 %) out of 700,000 reads. Therefore, we cut both the DRB and DQB fragments slightly (four first nucleotides of DQB fragments, eight last nucleotides of DRB fragments) to bring them back to the same length, which facilitated their alignment for further analyses and ensured that this recombination problem would not affect downstream analyses.

At this stage we recovered 706 unique DNA sequences for MHC-DRB in the range of 160–169 bp (excluding primers), and 827 for MHC-DQB, in the range of 162–169 bp. Among these, 555 DRB (79 %) and 680 DQB (82 %) sequences had not been previously described and were not retrieved from more than one individual. After discarding amplicons for which the total number of sequences was lower than 18 (15 for DRB and three for DQB), we obtained 654 individuals genotyped for DRB (711 amplicons including 53 duplicates and four triplicates, which represents 87 % of amplicons initially included in the sequencing process) and 643 (96 %) for DQB. The average (±SD) per amplicon coverage was 394 ± 429 reads for DRB and 394 ± 477 for DQB, with an extensive variation across amplicons since the range was 0–7042. Nevertheless, there was a positive correlation between the total number of reads and the number of distinct sequences per amplicon (Pearson’s correlation, DRB: *n* = 654 amplicons, *r* = 0.45, *P* < 10^−3^, DQB: *n* = 643 amplicons, *r* = 0.56, *P* < 10^−3^). This suggested that AAs appear more frequently when sequencing coverage is higher.

### Quality control

**Fig. 1:**
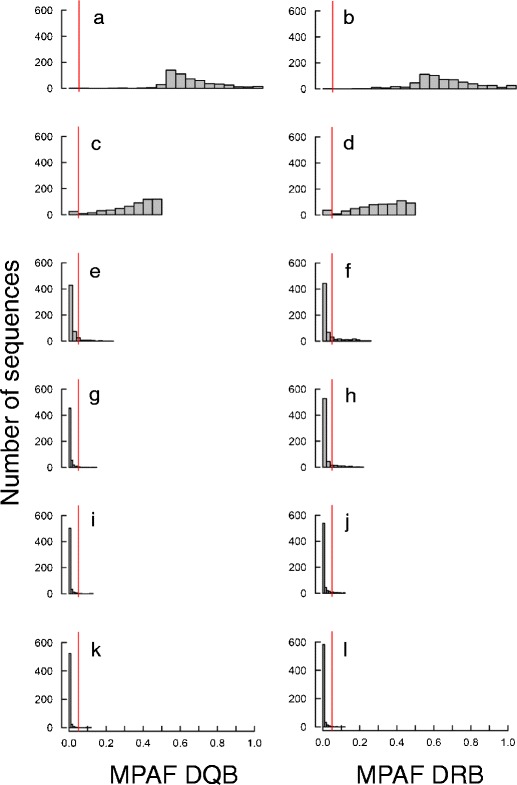
Distribution of mean per-amplicon frequency (*MPAF*) of the first to sixth variants found in the same amplicon for 707 DRB and 644 DQB amplicons. MPAF distributions of the most common allele (*MCA*) for DQB (**a**) and DRB (**b**), of the second (**c** DQB and **d** DRB), third (**e** DQB and **f** DRB), fourth (**g** DQB and **h** DRB), fifth (**i** DQB and **j** DRB) and sixth MCA (**k** DQB and **l** DRB) are shown. *Vertical line* stands for MPAF = 0.05

**Fig. 2:**
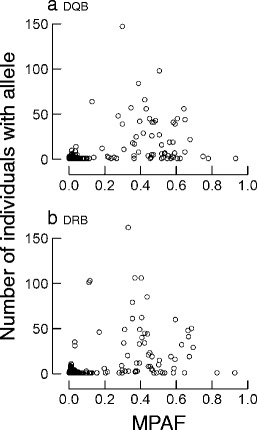
Plot of mean per-amplicon frequency (*MPAF*) in relation to allelic frequency (here indexed by the number of individuals carrying this allele) for 827 DQB (**a**) and 706 DRB (**b**) unique variant sequences, before the stepwise allelic validation procedure

We attempted to discriminate TA from AA through three complementary approaches. First, MPAF was found to be higher for the TMCA (DRB: mean ± SD = 0.48 ± 0.21; DQB: mean ± SD = 0.48 ± 0.19) than for the RA (DRB: mean ± SD = 0.04 ± 0.05; DQB: mean ± =0.03 ± 0.03). The bimodality of the distribution of MPAF of TMCA versus RA suggested that a threshold per-individual frequency value under which alleles are likely to represent AA approximates 5 % (Fig. 1). Second, for alleles with low MPAF, we compared their nucleotide sequence with those of the more common alleles retrieved from the same individuals, in order to check whether they could represent sequencing artefacts, namely a chimera of two more common sequences or a point-mutation variant of a more common sequence. Within 30 individuals possessing alleles with MPAF <0.01, we found that 26/30 (87 %) were AA. We then examined each individual possessing sequences with MPAF >0.03 and <0.05 in a similar fashion, and found that 27/30 (90 %) were AA. All sequences with MPAF <0.05 were eliminated on this basis. Finally, we examined each individual possessing sequences with MPAF >0.05 and <0.25 and found that 11/21 (52 %) were AA (which we eliminated). This confirmed a drastic decrease in the percentage of AA in sequences with MPAF higher than 0.05. As such, results from these two procedures converged in identifying 5 % as a good cut-off point for discriminating TA from AA based on MPAF in this dataset. Sequences remaining after this first screening — 61 DRB and 60 DQB alleles — were considered as TA. Finally, we examined the correlation between the MPAF and the number of individuals possessing a given allele before and after this screening step. The correlation was significant before allele sorting (Pearson’s correlation, DRB: *n* = 706 alleles, *r* = 0.49, *P* < 10^−3^; DQB: *n* = 827 alleles, *r* = 0.52, *P* < 10^−3^) and largely driven by the presence of alleles that had both low MPAF and low frequency (Fig. 2). As a result, it disappeared when retaining only TA (Pearson’s correlation, DRB: *r* = −0.09, *P* = 0.46; DQB: *r* = −0.16, *P* = 0.19).

Some individuals had more than two DRB (109 out of 654; 17 %) or DQB sequences (116 out of 643; 18 %) after this first screening. These additional sequences were characterized by low per-individual frequency (DQB: mean ± SD = 0.04 ± 0.03, median = 0.02; DRB: mean ± SD = 0.05 ± 0.05, median = 0.03). Consequently, we focused on those cases where additional sequences accounted for more than 5 % of the total number of reads to evaluate the probability of gene duplication. These included 41 (6 %) individuals with more than two DRB alleles and 35 (5 %) with more than two DQB alleles. Within this subset, the MPAF of the most common RA was 10 % (range for DRB: 5–20 %; range for DQB: 5–22 %). Fourteen individuals with more than two DRB alleles were re-genotyped, among which ten (71 %) were found to have only two alleles in the second run. Among the 41 individuals with more than two DRB alleles, 14 had a father and 13 had a mother genotyped. Among the 35 individuals with more than two DQB alleles, 20 had a father genotyped and 16 had a mother genotyped. They all inherited one allele (not more and not less) from each parent. In addition, alleles inherited from the parents systematically figured among the TMCA in offspring. As these results did not support the hypothesis of a possible duplication of either DRB or DQB in grey mouse lemurs, we adopted a conservative approach by considering individual genotypes to be the TMCA for the rest of the analyses.

Twenty-five individuals were found to be homozygous for DRB, and 20 for DQB genes. Fourteen were homozygous at both loci, suggesting true homozygosity at the haplotype level. Eleven homozygotes at DRB had both parents known, and seven for DQB loci. Homozygosity resulting from the inheritance of the same allele from both parents was confirmed in every case. Finally, six individuals who were found to be homozygous only at DRB and with 1 or 0 parents known were genotyped in duplicates. Five of them were confirmed as homozygotes.

We evaluated the accuracy of our genotyping in three validation steps. First, we compared the genotypes obtained by cloning and sequencing with the genotypes obtained by 454 sequencing for five individuals. These were perfectly congruent, once the chimeras had been eliminated from the resulting clones. Note that cloning generated an average of 17 % artefactual sequences (SD = 11 %, range = 9–35 %) retrieved from an average of 31 clones per individual genotyped (SD = 14 %, range = 20–56), which is even higher than 454 sequencing (given that the per-amplicon frequency of AAs is typically below 5 % — see above). Second, we evaluated the repeatability of individual genotypes by running duplicates for 53 individuals and triplicates for four individuals. There was 100 % repeatability for 47 heterozygous individuals, and one out of six homozygotes was found to be heterozygote. Third, we systematically checked patterns of inheritance in our sample. 239 mother–offspring dyads were available for both DQB and DRB, 262 father–offspring dyads for DQB and 256 for DRB. The percentage of mismatches was lower than 2 % in every case, and all mismatches occurred at both loci for a given individual, thus possibly resulting from a low error rate in parentage assignments. Overall, our genotyping proved highly accurate.

### Patterns of *Mimu*-DRB and DQB variability

**Fig. 3:**
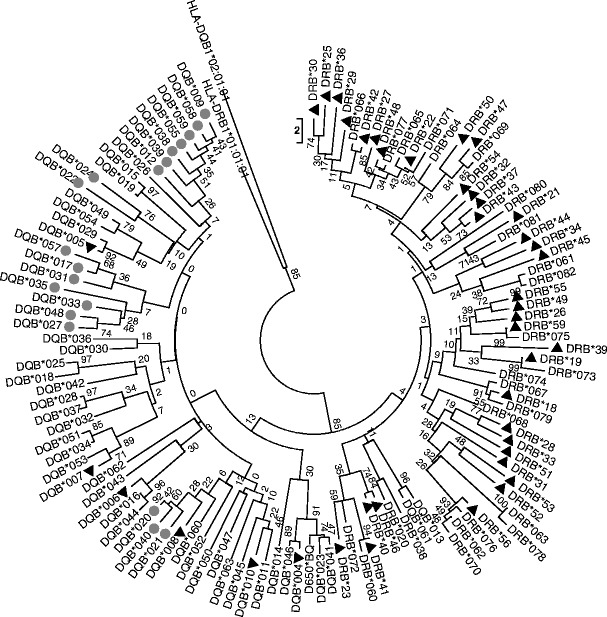
Phylogenetic tree of the *Mimu-*DQB and *Mimu-*DRB sequences observed and described in this study. Previously published sequences are marked with a *black triangle* —others are new. *Mimu*-DQB sequences exhibiting a 6-bp deletion are marked with *plain grey circles*. The tree configuration was derived from nucleotide sequences using the neighbour-joining method implemented in MEGA. *HLA-*DQB1*02 (accession number: FR798950.1) and *HLA-*DRB1*01 (accession number: FN821964.1) sequences are shown for comparative purposes. Accession numbers and nucleotide sequence of the *Mimu-*DQB and *Mimu-*DRB alleles are presented in Appendix

We found high genetic variability in the study population, with a total of 61 DRB (163 bp) and 60 DQB (163 or 169 bp) alleles among 654 and 643 individuals, respectively. We identified 20 and 53 new DRB and DQB alleles, respectively. Sequence information (including the nucleotide sequences of the alleles that were not published) and accession numbers are summarised in the Appendix (Tables 4, 5, and Fig. 7). *Mimu-*DRB and -DQB sequences show wide-ranging levels of divergence with 56 (34 %) variable sites in the DRB sequences and 45 (27 %) in the DQB sequences. There was an average of 14.6 ± 2.2 (DRB) and 15.2 ± 2.4 (DQB) nucleotide differences between sequences. Nineteen DQB sequences showed an insertion of six nucleotides (two codons) compared to other DQB and DRB variants. Each sequence had a unique amino-acid sequence and the absence of stop codons suggests that all nucleotide sequences could encode functional proteins. In DRB sequences, 29 (52 %) variable sites were identified on a 56-amino acid sequence. In DQB, 25 and 26 (45 %) variable sites were identified on a 56- and 58- (with insertion) amino acid sequence, respectively. Analyses of amino acid differences between sequences revealed an average of 10.6 ± 2.0 differences for DRB and 10.4 ± 1.8 for DQB sequences, respectively. DRB and DQB nucleotide sequences clustered in two clearly separated phylogenetic lineages (Fig. 3). At the population level, allelic frequencies varied widely, from 0.1 to 21 % (median 2 %) for both DQB and DRB.

### Signatures of historical selection

**Fig. 4:**
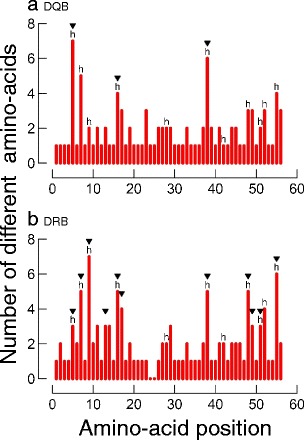
Amino-acid variation plot for **a** DQB and **b** DRB alleles. Human antigen-binding sites (*ABS*) are indicated with the letter ‘h’, whereas positively selected sites (*PSS*) are indicated with black triangles. In the DRB plot there are no amino acids between the positions 24–25, because these alleles were only 163 bp long, whereas 19 DQB alleles were 169 bp long, with an insertion of two codons at positions 24–25

PSS were identified in *Mimu*-DRB and -DQB sequences using a maximum-likelihood analysis. The significant deviation of the LRT statistic from a *χ* ^2^ distribution (DRB: *χ* ^2^ = 36.57, *df* = 2, *P <* 0.001, DQB: *χ* ^2^ = 81.14, *df* = 2, *P <* 0.001) allowed rejection of the null model assuming neutral evolution (M7) in favour of a model allowing for a class of sites subjected to diversifying selection (M8) for both loci (Table 2). At the DRB locus, 16 sites were detected as PSS by this approach, and 11 were statistically significant (*P <* 0.05) according to Bayes empirical analysis. At the DQB locus, three sites were detected as PSS by this approach and all were statistically significant. The non-significant PSSs were excluded from subsequent analyses. Eight PSS were identical to the ABS defined by homology with HLA-DRB (Bondinas et al. 2007) (Fig. 4). The remaining PSS (amino acid positions 13, 17 and 49) were situated within a distance of three, two and one amino acid(s) of an ABS, respectively. In DQB, all three PSSs corresponded to the ABS defined by homology with HLA-DQB (Fig. 4). Three human ABS (positions 28, 42, 52) were not identified as PSS in *Mimu*-DRB, and nine in *Mimu*-DQB (positions 7, 9, 28, 38, 42, 48, 51, 52, 55). Finally, estimated values of *d*_S_, *d*_N_ through the evolutionary pathways method confirmed results of the maximum likelihood analysis through increased values of ω at both ABS and PSS, relatively to non-ABS and non-PSS, respectively (Table 3).

**Table 2:** Evaluation of the goodness of fit for different models of codon evolution and estimated parameter values

Model	LnL	AIC	∆AIC	Parameters
**Mimu-DRB**				
M0 — one ω	−1,662.8	3,327.7	306.9	*ω* = 0.82
M7 — nearly neutral with β	−1,517.7	3,055.4	34.8	
M8 — positive selection with *β* (*ω*_0_ ≤ 1, *ω*_1_ > 1)	−1,499.4	3,020.8	Best	*p*_0_ = 0.75, *p*_1_ = 0.25, *ω*_1_ = 2.92
**Mimu-DQB**				
M0 — one *ω*	−1,773.0	3,548.0	433.1	*ω* = 0.65
M7 — nearly neutral with *β*	−1,587.0	3,194.1	79.2	
M8 — positive selection with *β* (*ω*_0_ ≤ 1, *ω*_1_ > 1)	−1,546.5	3,114.9	Best	*p*_0_ = 0.94, *p*_1_ = 0.05, *ω*_1_ = 5.51

*ω* — dN/dS; nearly neutral with beta — for all sites *ω* ≤ 1 and the beta distribution approximates *ω* variation; positive selection — a proportion of sites evolves with *ω* > 1; *p*_0_ — proportion of sites with *ω* ≤ 1, *p*_1_ — proportion of positively selected sites (*ω* > 1), *ω*_1_ — estimated value of *ω* for sites under positive selection

*AIC* Akaike information criterion, *ΔAIC* difference between the value of the AIC of a given model and the best model

**Table 3:** Results of the evolutionary pathways method (Nei and Gojobori 1986) to estimate values of *d*_S_, *d*_N_ and their standard errors for antigen (ABS) or non-antigen (non-ABS) binding sites defined by homology with HLA (Bondinas et al. 2007) and for codon sites identified as positively selected sites (PSS) or as non-positively selected sites (non-PSS) within the 61 Mimu-DRB and the 60 Mimu-DQB sequences

Positions	*n*	*d*_N_	*d*_S_	*ω*	*Z* (*P*)
Mimu-DRB					
ABS	11	0.49 ± 0.09	0.07 ± 0.04	6.84	5.22 (0.00)
Non-ABS	45	0.04 ± 0.01	0.03 ± 0.02	1.30	0.50 (0.62)
PSS	11	0.52 ± 0.06	0.12 ± 0.08	4.20	4.08 (0.00)
Non-PSS	45	0.04 ± 0.01	0.02 ± 0.01	2.08	1.32 (0.19)
All	54	0.12 ± 0.02	0.04 ± 0.02	3.02	2.87 (0.05)
Mimu-DQB					
ABS	11	0.37 ± 0.10	0.01 ± 0.01	26.84	3.33 (0.01)
Non-ABS	45	0.07 ± 0.02	0.05 ± 0.02	1.37	0.71 (0.48)
PSS	3	0.59 ± 0.27	0.03 ± 0.01	22.17	2.12 (0.04)
Non-PSS	51	0.04 ± 0.02	0.10 ± 0.02	0.43	2.05 (0.04)
All	54	0.12 ± 0.03	0.04 ± 0.02	2.82	2.51 (0.01)

*n* — number of codons in each category; *ω* — *d*_N_/*d*_S_; *P —* probability that *d*_N_ and *d*_S_ are similar using a *Z*-test of selection

### Patterns of MHC class II diversity at the population level

**Fig. 5:**
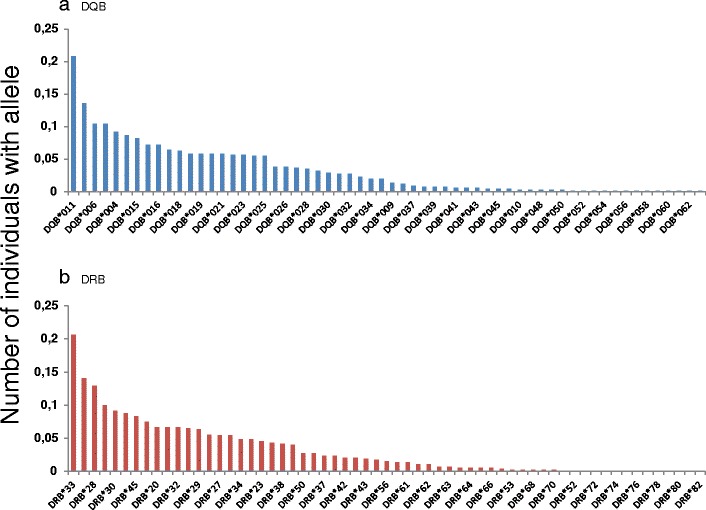
Allelic distribution for **a** DQB alleles and **b** DRB alleles in the study population

**Fig. 6:**
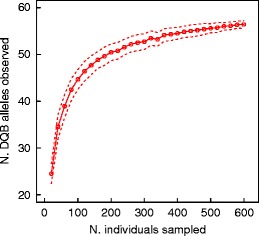
Estimation of population allelic richness through a resampling procedure counting the number of DQB alleles detected in relation to the number of individuals sampled. The *dotted lines* indicate the standard deviation of the estimated mean. The corresponding pattern is not shown for DRB, because it looks very similar

**Fig. 7:**
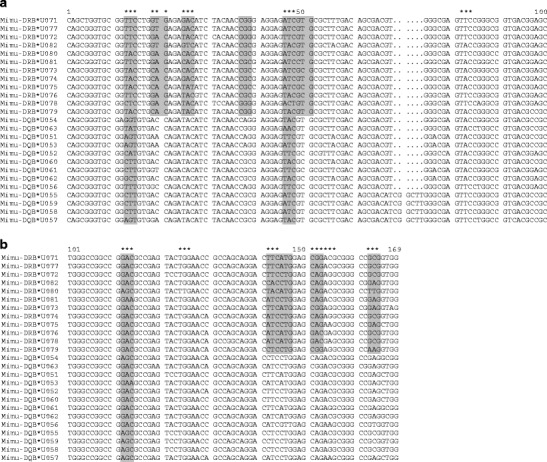
Alignment of the DRB and DQB alleles retrieved from no more than one individual. Antigen-binding sites (ABS) are indicated with *, whereas positively selected sites (PSS) are highlighted in *grey*. Alignment gaps are indicated by *dots*

Both genes showed very similar allelic distribution patterns (Fig. 5) due to their physical linkage translating into a high genotypic disequilibrium (*χ* ^2^ = 7,643.0, *df* = 2, *P <* 0.001). The estimated frequency of null alleles was extremely low for both loci (DRB: 0.0 %; DQB: 0.6 %), and neither DRB nor DQB locus showed a significant heterozygote excess (DRB: Fis = −0.007, *P* = 0.14; DQB: Fis = −0.004, *P* = 0.49). Plotting the average number of alleles detected for a given sampling effort showed that estimating allelic richness required sampling more than 100 individuals in this population, as the curve is quasi-linear below that threshold (Fig. 6). A minimum of 200 individuals would allow detecting an elbow in the shape of the relationship, and the asymptotic trend suggests that most alleles have now been described in this population.

## Discussion

We used large-scale MHC class II genotyping in more than 600 grey mouse lemurs using 454 sequencing technology. Several validation steps demonstrated the accuracy of our genotyping. A total of 61 DRB (including 20 new) and 60 (including 53 new) DQB alleles were identified; tests of historical selection implied this polymorphism was mainly maintained by balancing selection in both the DRB and DQB locus, though results also suggested that the strength of selection might vary across these regions. Lastly, we examined basic patterns of MHC class II diversity at the population level. Here, we first comment on the potential of MHC genotyping using 454 sequencing, highlighting the most sensitive steps in our experiment. We then consider how our findings extend prior knowledge regarding MHC class II polymorphisms in the grey mouse lemur.

### Optimisation of 454 sequencing in the study system

Our protocol consisted in simultaneously amplifying DRB and DQB amplicons from 95 individuals in a same 454-sequencing run, returning around 110,000 reads. A first complication was caused by the simultaneous amplification of both genes. Despite a very comparable length of the amplified fragments, our first run yielded almost twice as many DQB as DRB sequences. The reasons behind this problem remain unclear, but adding a double amount of DRB compared to DQB in the sequencing mix of the next runs redressed this bias. This observation reveals the potential difficulty of adjusting the coverage when sequencing several genes amplified with distinct primer pairs in a same library. It might then be useful to optimize the coverage with a trial run using the 454 Junior system, before switching, for instance, to a 454 FLX sequencer to genotype the full sample. Once optimised, our methods provided an average of 400 reads per individual for the final analysis. Despite conducting a careful individual concentration check for each amplicon, there was an extensive within-run between-individual variance in the number of reads, as well as a significant (though less substantial) variance in the total number of reads per run. This extensive between-amplicon variance was probably a consequence of our high coverage, as the variance typically increases with the mean of a distribution. Other projects frequently report a lower variance in their coverage, but also a higher proportion of non-genotyped individuals (Babik et al. 2009; Galan et al. 2010; Sepil et al. 2012; Zagalska-Neubauer et al. 2010).

Another complication arising from sequencing several genes using distinct primers was the occurrence of recombinant sequences (chimeras) associating the DRB forward primer with the DQB reverse primer. This problem likely results from a relatively high level of sequence similarity between DRB and DQB sequences, and might be prevalent when working on MHC genes, since the occurrence of chimeras has long plagued MHC genotyping, consistently affecting cloning and amplification of MHC sequences (Babik 2010).

### Quality control procedure

The main challenge of applying next-generation sequencing technologies to MHC genotyping probably lies in the high frequency of sequencing errors (Babik 2010; Babik et al. 2009; Zagalska-Neubauer et al. 2010). Procedures aimed at discriminating TAs from AAs have already been proposed by several authors (Babik et al. 2009; Galan et al. 2010; Zagalska-Neubauer et al. 2010), and constituted the basis of our stepwise variant validation procedure. We applied an additional step, based on the fact that the examined loci were non-duplicated in our study species and that each individual should display no more than two TAs.

A significant proportion of individuals (15–20 %) exhibited more than two TAs after this initial quality control, which had presumably eliminated AAs. These sequences were unlikely to be misassigned due to a sequencing error in the individual tag, given the probability of such misassignment. The lack of vertical transmission of such additional sequences among parent–offspring dyads does not support the hypothesis of polymorphism in the gene copy number, and led us to use the two most common variants (within an amplicon) as the final genotype for each individual. We cannot exclude that some of these sequences result from the amplification of paralogues or pseudogenes, but this is not supported by the fact that such additional sequences were found to be TMCA in other individuals. This observation rather pointed to the occurrence of contaminations across amplicons, which might have occurred across runs since we used several consecutive small runs (with a GS Junior sequencer) rather than a large one (with a FLX). Nevertheless, contaminations were probably easier to detect in our system than elsewhere, because the number of copies expected for each individual was known, and an extensive number of parent–offspring dyads were genotyped. It might thus represent an under-appreciated problem in MHC genotyping using NGS, especially if the number of duplicates is low. Setting a minimum MPAF threshold to eliminate variants occurring at very low frequency within amplicons — even if they represent TA — might represent a good precaution.

Finally, the quality of the final genotypes was validated through three procedures: (1) comparing genotypes obtained by 454 sequencing and by cloning for five individuals, (2) running duplicates, and (3) comparing parent–offspring dyads. Note that error rates estimated through the number of parent–offspring mismatches are rarely communicated by studies subsequently testing for MHC-biased reproduction, while pedigree information is often available. This would help to evaluate the extent to which genotyping quality might affect downstream hypothesis testing.

### Grey mouse lemur MHC class II variability

We identified a total of 61 DRB alleles (20 of which were new) and 60 DQB alleles (53 of which were new). Combined with the low number of MHC class II genes (which are non-duplicated), this considerable allelic polymorphism contrasts with the organisation of the homologous region in Old World monkeys, characterized by a low degree of allelic polymorphism and a high degree of flexibility in the configuration of the region (de Groot et al. 2008; Doxiadis et al. 2010) as well as with the New World monkeys which have duplicated DRB genes with low polymorphism (Bontrop 2006). The extent of allelic polymorphism probably also reflects the outbred nature and the large size of our study population. Examining the effect of the sampling scheme — number of individuals genotyped — on the number of alleles detected suggests that we have now identified most allelic variation in both genes at the population level. Such an analysis is potentially useful for future attempts to understand patterns of MHC diversity across populations or species, which might be better able to control for methodological variation among surveys.

Despite their relative similarity, DRB and DQB sequences clustered in two distinct groups in the phylogenetic analysis. Only two DQB alleles (*Mimu*-DQB*013 and *Mimu*-DQB*U61) clustered with DRB alleles. A BLAST of these alleles yielded *Mimu*-DQB*002, a known allele previously isolated from the same population (Averdam et al. 2011) as their closest match. These sequences are thus correctly identified as DQB alleles, despite their structural similarity to DRB lineages. Given the tight linkage, shared origin and functional similarity of DRB and DQB (Trowsdale 1993), it might not be surprising that the clustering of DQB and DRB alleles is not perfect when considering a short fragment concentrating the majority of ABS. In addition, a six-nucleotide insertion characterizing 19 DQB sequences resulted in fragment length polymorphism within DQB sequences. Although these alleles tended to cluster together, they did not form a distinct lineage. This may suggest that they are still under strong selection, and that the deletion does not alter the protein function. Deleterious mutations may be more strongly counterselected in grey mouse lemurs where DRB and DQB loci are not duplicated, than in species with multiple copies, where the (partial) loss of function of one copy may be compensated by others. Indeed, no evidence for stop codons, nor for divergent allelic lineages evoking pseudogenes, had been found in a total of 63 DQB and 82 DRB alleles detected in this population (see Averdam et al. 2011; Schwensow et al. 2008 for the description of the DQB and DRB alleles non retrieved in this study, respectively).

Patterns of nucleotide substitutions occurring on both sequences revealed signatures of historical selection, through the occurrence of a number of PSS. At DRB, nine PSS occurred at, or close to, the antigen biding sites (ABS, inferred by comparison with *HLA*-DRB). By contrast, we only detected three PSS in DQB sequences. Given that the power of the analyses was identical — since we had the same number of sequences — natural selection acting on the DQB locus might have been weaker compared to DRB. Yet, estimates of *d*_N_/*d*_S_ ratios do not particularly support this possibility, as both genes appear to show intense traces of selection. It is possible that the protein structure differs among loci, with a lower number of functionally more important sites in DQB, but this interpretation is speculative. It is also important to note that selection pressures operating on both regions are not independent, since *Mimu*-DRB and -DQB genes are located on a same chormosome, and separated by about 100 kb (Averdam et al. 2011). The tight linkage disequilibrium observed here therefore mirrors their physical linkage, suggesting that there is no recombination hotspot between them. As such, balancing selection acting on one locus contributes to shape polymorphism at the other. A tight physical association between DRB and DQB is well conserved across mammals including primates (Bontrop et al. 1999; Kelley et al. 2005) and probably reflects their functional similarity, as there is a great deal of flexibility in the class II genes used for antigen presentation across mammals (Trowsdale 1993).

Finally, the two loci did not present any heterozygote excess or deficiency, which confirms the low frequency of genotyping errors, as well as the absence of major population subdivision or genetic drift. This might also suggest an absence of viability selection for heterozygous individuals or particular alleles, together with random mating in the study population, or alternatively failure to detect signatures of selection at the population level, despite evidence of historical selection at the molecular level. Previous work in this population suggests that natural selection still shapes patterns of MHC diversity, as MHC genes impact reproduction (Schwensow et al. 2008) and parasite resistance (Schwensow et al. 2010). Nevertheless, reported mating biases consisted in a choice for individuals that had different MHC supertypes (functional categories of MHC sequences) and divergent allelic combinations (a measure of intra-individual diversity) rather than different sequences (Schwensow et al. 2008), while parasite-driven selection favours some MHC alleles over others, as well as divergent allelic combinations (Schwensow et al. 2010). These patterns might not necessarily translate into a heterozygote excess. In addition, theory indicates that deviations from HWE is a poor indicator of natural selection in the wild, as detecting strong viability selection might imply using a sample size of more than 1,000 individuals (Lachance 2009). Finally, using HWE as a conclusive tool to detect selection would require taking into account the age structure of the population (Alvarez 2008), which was beyond the scope of this study.

## Conclusions

In line with previous work on the MHC class II genes in the grey mouse lemur we found extensive allelic diversity at both DRB and DQB genes following a large-scale, high-resolution genotyping effort. Both loci were non-duplicated and tightly linked, but evidence for balancing selection was strongest on DRB genes. It will now be interesting to compare the strength of contemporary selection operating at both loci by investigating MHC-associated fitness effects in our study population. Our results generally emphasize the important potential of 454 technology for attempts to understand how parasite-mediated and sexual selection shape and maintain host immune genetic variation in nature. This will allow genotyping entire populations across consecutive generations, finally providing sample sizes that are adequate for these research questions. The power and promises of this approach will nevertheless be conditional upon establishing strict quality control of the final genotyping dataset, ideally by combining several methods following our example.

## Appendix

**Table 4:** Description of MHC-DQB sequences from *Microcebus murinus* observed or described in this study

Allele name	*N* ind.	*N* reads	Length (bp)	New?	Genbank accession number/nucleotide sequence (5′–3′)
Mimu-DQB*004	60	10,902	163	no	HQ222948.1
Mimu-DQB*005	38	11,209	163	no	HQ222949.1
Mimu-DQB*006	68	4,199	163	no	HQ222950.1
Mimu-DQB*007	25	3,452	163	no	HQ222951.1
Mimu-DQB*008	47	8,074	163	no	HQ222952.1
Mimu-DQB*009	9	430	169	no	HQ222953.1
Mimu-DQB*010	2	565	163	no	HQ222954.1
Mimu-DQB*011	135	16,041	163	yes	HE801914
Mimu-DQB*012	88	13,497	169	yes	HE801915
Mimu-DQB*013	68	9,688	163	yes	HE801916
Mimu-DQB*014	56	7,043	163	yes	HE801917
Mimu-DQB*015	53	16,961	163	yes	HE801918
Mimu-DQB*016	47	4,275	163	yes	HE801919
Mimu-DQB*017	42	12,215	169	yes	HE801920
Mimu-DQB*018	41	6,896	163	yes	HE801921
Mimu-DQB*019	38	9,633	163	yes	HE801922
Mimu-DQB*020	38	4,252	169	yes	HE801923
Mimu-DQB*021	38	8,113	169	yes	HE801924
Mimu-DQB*022	37	9,690	169	yes	HE801925
Mimu-DQB*023	37	5,263	163	yes	HE801926
Mimu-DQB*024	36	6,093	169	yes	HE801927
Mimu-DQB*025	36	9,911	163	yes	HE801928
Mimu-DQB*026	25	3,640	163	yes	HE801929
Mimu-DQB*027	24	4,070	169	yes	HE801930
Mimu-DQB*028	23	2,924	163	yes	HE801931
Mimu-DQB*029	21	5,219	163	yes	HE801932
Mimu-DQB*030	19	4,742	163	yes	HE801933
Mimu-DQB*031	18	2,443	169	yes	HE801934
Mimu-DQB*032	18	4,209	163	yes	HE801935
Mimu-DQB*033	15	1,971	169	yes	HE801936
Mimu-DQB*034	13	2,596	163	yes	HE801937
Mimu-DQB*035	13	1,919	169	yes	HE801938
Mimu-DQB*036	8	1,880	163	yes	HE801939
Mimu-DQB*037	6	1,574	163	yes	HE801940
Mimu-DQB*038	5	405	169	yes	HE801941
Mimu-DQB*039	5	705	169	yes	HE801942
Mimu-DQB*040	5	1,076	169	yes	HE801943
Mimu-DQB*041	4	386	163	yes	HE801944
Mimu-DQB*042	4	911	163	yes	HE801945
Mimu-DQB*043	4	461	163	yes	HE801946
Mimu-DQB*044	3	243	163	yes	HE801947
Mimu-DQB*045	3	388	163	yes	HE801948
Mimu-DQB*046	3	542	163	yes	HE801949
Mimu-DQB*047	2	106	163	yes	HE801950
Mimu-DQB*048	2	217	169	yes	HE801951
Mimu-DQB*049	2	1,517	163	yes	HE801952
Mimu-DQB*050	2	56	163	yes	HE801953
Mimu-DQB*U051	1	152	163	yes	CAGCGGGTGCGGAGTGTGAACAGATACATCTACAACC
					AGGAGGAGTTCGTGCGCTACGACAGCGACGTGGACGA
					GTACCGGGCCGTGACGCCGCTGGGCCGGCCGGACGCC
					GAGTCCTGGAACCGCCAGCAGGACTTCATGGAGCGGA
					CGCGGGCCGAGCTGG
Mimu-DQB*U052	1	134	163	yes	CAGCGGGTGCGGCATGTGACCAGATACATCTACAACC
					GGGAGGAGTTCGCGCGTTTCGACAGCGACGTGGGCCA
					GTACCGGGCCGTGACGGAGCTGGGCCGGCCGGACGCC
					GAGTACTGGAACAGCCAGCAGGACTTCCTGGAGCAGA
					CGCGGGCCGAGCTGG
Mimu-DQB*U053	1	255	163	yes	CAGCGGGTGCGGAGTGTGAACAGATACATCTACAACC
					AGGAGGAGATCGTGCGCTACGACAGCGACGTGGACGA
					GTACCGGGCCGTGACGCCGCTGGGCCGGCCGGAAGCC
					GAGTACTGGAACCGCCAGCAGGACATCATGGAGCGGA
					CGCGGGCCGAGCTGG
Mimu-DQB*U054	1	116	163	yes	CAGCGGGTGCGAGGTGTGACCAGATACATCTACAACC
					AGGAGGAGTACGTGCGCTTCGACAGCGACGTGGGCGA
					GTACCGGGCGGTGACGCCGCTGGGCCGGCCGAGCGCC
					GAGTACTGGAACAGCCAGCAGGACCTCCTGGAGCAGA
					CGCGGGCCGAGGCGG
Mimu-DQB*U055	1	427	169	yes	CAGCGGGTGCGGCTTGTGACCAGATACATCTACAACC
					GCGAGGAGATCGTGCGCTTCGACAGCGACATCGGCTT
					GGGCGAGTTCCGGGCGGTGACGCCGCTGGGCCGGCCG
					AGCGCCGAGTCCTGGAACCGCCAGCAGGACCTCCTGG
					AGCAGACGCGGGCCGCGGTGG
Mimu-DQB*U056	1	319	163	yes	CAGCGGGTGCGGTTTGTGGCCAGACACATCTACAACC
					AGGAGGAGTTCGCGCGCTTCGACAGCGACGTGGGCGA
					GTTCCTGGCCGTGACGGAGCTGGGCCGGCCGGACGCC
					GAGTACTGGAACCGCCAGCAGGACATCGTTGAGCAGA
					AGCGGGCCGTGGTGG
Mimu-DQB*U057	1	24	169	yes	CAGCGGGTGCGGAGTGTGGACAGATACATCTACAACC
					GCGAGGAGTACGTGCGCTTCGACAGCGACATCGGCTT
					GGGCGAGTTCCTGGCCGTCACGCCGCTGGGCCGGCCG
					AGCGCCGAGTACTGGAACAGCCAGCAGGACATCCTGG
					AGCAGAAGCGGGCCGAGCTGG
Mimu-DQB*U058	1	173	169	yes	CAGCGGGTGCGGCTTGTGACCAGATACATCTACAACC
					GCGAGGAGATCGTGCGCTTCGACAGCGACATCGGCTT
					GGGCGAGTTCCGGGCCGTGACGCCGCTGGGCCGGCCG
					AGCGCCGAGTCCTGGAACCGCCAGCAGGACCTCCTGG
					AGCAGAGGCGGGCCGCGGTGG
Mimu-DQB*U059	1	55	169	yes	CAGCGGGTGCGGCTTGTGACCAGATACATCTACAACC
					GCGAGGAGATCGTGCGCTTCGACAGCGACATCGGCTT
					GGGCGAGTTCCGGGCGGTGACGCCGCTGGGCCGGCCG
					AGCGCCGAGTCCTGGAACCGCCAGCAGGACCTCCTGG
					AGCAGAGGCGGGCCGCGGTGG
Mimu-DQB*U060	1	57	163	yes	CAGCGGGTGCGGCTTGTGACCAGATACATCTACAACC
					GCGAGGAGTACGCGCGCTACGACAGCGACGTGGGCGA
					GTACCGGGCCGTGACGCCGCTGGGCCGGCCGGACGCC
					GAGTACTGGAACCGCCAGCAGGACTTCCTGGAGCAGA
					CGCGGGCCGAGCTGG
Mimu-DQB*U061	1	92	163	yes	CAGCGGGTGCGGCTTGTGGTCAGACACATCTACAACC
					GCGAGGAGTTCGCGCGCTTCGACAGCGACGTGGACGA
					GTACCGGGCGGTGACGGAGCTGGGCCGGCCGGACGCC
					GAGTACTGGAACCGCCAGCAGGACTTCATGGAGCAGA
					GGCGGGCCGAGGCGG
Mimu-DQB*U062	1	133	163	yes	CAGCGGGTGCGGGCTGTGACCAGATACATCTACAACC
					GCGAGGAGTACGCGCGCTACGACAGCGACGTGGACGA
					GTACCGGGCCGTGACGGAGCTGGGCCGGCCGGACGCC
					GAGTACTGGAACCGCCAGCAGGACATCATGGAGCAGA
					CGCGGGCCGAGGCGG
Mimu-DQB*U063	1	508	163	yes	CAGCGGGTGCGGTATGTGACCAGATACATCTACAACC
					GGGAGGAGAACGTGCGCTTCGACAGCGACGTGGGCGA
					GTACCTGGCCGTGACGCCGCTGGGCCGGCCGGACGCC
					GAATACTGGAACAGCCAGCAGGACATCCTGGAGCGGA
					CGCGGGCGGAGGTGG

The number of individuals (*N* ind.) from which each sequence was retrieved, as well as the total number of reads (across runs and amplicons) for each sequence is given. The nucleotide sequence of new sequences retrieved from only one individual is shown in this table; such sequences were not submitted to public repository to ensure the storage of high quality sequences in such databases

**Table 5:** Description of MHC-DRB sequences from *M. murinus* observed or described in this study

DRB allele	*N* amp.	*N* reads	Length (bp)	New?	Genbank accession number/nucleotide sequence
Mimu-DRB*18	101	15,821	163	no	EU137062.1
Mimu-DRB*19	72	11,907	163	no	EU137063.1
Mimu-DRB*20	48	6,932	163	no	EU137064.1
Mimu-DRB*21	31	3,693	163	no	EU137065.1
Mimu-DRB*22	48	5,619	163	no	EU137066.1
Mimu-DRB*23	33	4,730	163	no	EU137067.1
Mimu-DRB*25	4	1,080	163	no	EU137069.1
Mimu-DRB*26	3	289	163	no	EU137070.1
Mimu-DRB*27	39	9,123	163	no	EU137071.1
Mimu-DRB*28	93	13,558	163	no	EU137072.1
Mimu-DRB*29	46	13,279	163	no	EU137073.1
Mimu-DRB*30	66	10,127	163	no	EU137074.1
Mimu-DRB*31	5	753	163	no	EU137075.1
Mimu-DRB*32	48	15,472	163	no	EU137076.1
Mimu-DRB*33	148	16,221	163	no	EU137077.1
Mimu-DRB*34	35	5,850	163	no	EU137078.1
Mimu-DRB*36	63	14,297	163	no	EU137080.1
Mimu-DRB*37	17	4,546	163	no	EU137081.1
Mimu-DRB*38	30	7,888	163	no	EU137082.1
Mimu-DRB*39	54	7,208	163	no	EU137083.1
Mimu-DRB*40	40	5,528	163	no	EU137084.1
Mimu-DRB*41	47	2,470	163	no	EU137085.1
Mimu-DRB*42	15	4,288	163	no	EU137086.1
Mimu-DRB*43	14	3,296	163	no	EU137087.1
Mimu-DRB*44	29	7,933	163	no	EU137088.1
Mimu-DRB*45	60	15,058	163	no	EU137089.1
Mimu-DRB*46	39	8,776	163	no	EU137090.1
Mimu-DRB*47	15	2,450	163	no	EU137091.1
Mimu-DRB*48	8	1,322	163	no	EU137092.1
Mimu-DRB*49	1	184	163	no	EU137093.1
Mimu-DRB*50	20	3,000	163	no	EU137094.1
Mimu-DRB*51	10	1,624	163	no	EU137095.1
Mimu-DRB*52	1	329	163	no	EU137096.1
Mimu-DRB*53	2	97	163	no	EU137097.1
Mimu-DRB*54	13	1,275	163	no	EU137098.1
Mimu-DRB*55	35	3,652	163	no	EU137099.1
Mimu-DRB*56	11	1,510	163	no	EU137100.1
Mimu-DRB*59	17	1,839	163	no	EU137103.1
Mimu-DRB*60	20	4,035	163	yes	HE801954
Mimu-DRB*61	10	1,031	163	yes	HE801955
Mimu-DRB*62	8	923	163	yes	HE801956
Mimu-DRB*63	5	252	163	yes	HE801957
Mimu-DRB*64	4	591	163	yes	HE801958
Mimu-DRB*65	4	288	163	yes	HE801959
Mimu-DRB*66	4	336	163	yes	HE801960
Mimu-DRB*67	2	42	163	yes	HE801961
Mimu-DRB*68	2	397	163	yes	HE801962
Mimu-DRB*69	2	372	163	yes	HE801963
Mimu-DRB*70	2	231	163	yes	HE801964
Mimu-DRB*U071	1	9	163	yes	CAGCTGGTGCGGTTCCTGGTGAGAGACATCTACAACC
					GGGAGGAGATCGTGCGCTTTGACAGCGACGTGGGCGA
					GTTCCGGGCGGTGACGGAGCTGGGCCGGCCGGACGCC
					GAGTACTGGAACCGCCAGCAGGACTTCATGGAGCGGA
					CGCGGGCCGCGGTGG
Mimu-DRB*U072	1	248	163	yes	CAGCGGGTGCGGCTCCTGGACAGATACATCCACAACG
					GGGAGGAGTTCGTGCGCTTCGACAGCGACGTGGGCGA
					GTTCCGGGCGGTGACGGAGCTGGGCCGGCCGGACGCC
					GAGTCCTGGAACCGCCAGCAGGACTTCCTGGAGCAGA
					GGCGGGCCGCGGTGG
Mimu-DRB*U073	1	9	163	yes	CAGCGGGTGCGGTACCTGCACAGACACATCTACAACC
					GCCAGGAGATCGCGCGCTTCGACAGCGACGTGGGCGA
					GTACGGGGCGGTGACGGAGCTGGGCCGGCCGGACGCC
					GAGTACTGGAACCGCCAGCAGGACTTCATGGAGCGGA
					GGCGGGCGGAGGTAG
Mimu-DRB*U074	1	125	163	yes	CAGCGGGTGCGGTACCTGCACAGAGACATCTACAACC
					GCGAGGAGATCGTGCGCTTCGACAGCGACGTGGGCGA
					GTACCGGGCGGTGACGGAGCTGGGCCGGCCGGACGCC
					GAGTACTGGAACAGCCAGCAGGACATCCTGGAGCAGA
					GGCGGGCCGCGGTGG
Mimu-DRB*U075	1	9	163	yes	CAGCGGGTGCGGTACCTGCACAGATATATCTACAACC
					GCCAGGAGATCGCGCGCTTCGACAGCGACGTGGGCGA
					GTACCGGGCGGTGACGGAGCTGGGCCGGCCGGACGCC
					GAGTACTGGAACCGCCAGCAGGACATCCTGGAGCAGA
					AGCGGGCCGAGCTGG
Mimu-DRB*U076	1	113	163	yes	CAGCGGGTGCGGTTCCTGGAGAGATACGTCTACAACC
					GCGAGGAGTACGCGCGCTTCGACAGCGACGTGGGCGA
					GTTCCGGGCGGTGACGGAGCTGGGCCGGCCGGACGCC
					GAGTACTGGAACAGCCAGCAGGACATCATGGAGGACG
					AGCGGGCCGCGGTGG
Mimu-DRB*U077	1	165	163	yes	CAGCGGGTGCGGTTCCTGGTGAGAGACATCTACAACC
					GGGAGGAGATCGTGCGCTTCGACAGCGACGTGGGCGA
					GTTCCGGGCGGTGACGGAGCTGGGCCGGCCGGACGCC
					GAGTACTGGAACCGCCAGCAGGACTTCATGGAGCAGA
					CGCGGGCCGCGGTGG
Mimu-DRB*U078	1	43	163	yes	CAGCGGGTGCGGCTCCTGGACAGATACATCTCCAACG
					GGGAGGAGACTGTGCGCTTCGACAGCGACGTGGGCGA
					GTTCCAGGCGGTGACGGAGCTGGGCCGGCCGGACGCC
					GAGTACTGGAACAGCCGGCAGGACATCATGGAGGACG
					AGCGGGCCGCGGTGG
Mimu-DRB*U079	1	86	163	yes	CAGCGGGTGCGGTACCTGCACAGATACATCTACAACC
					GGGAGGAGTACGTGCGCTTCGACAGCGACGTGGGCGA
					GTACCGGGCGGTGACGCCGCTGGGCCGGCCGGACGCC
					GAGTACTGGAACAGCCAGCAGGACCTCCTGGAGCGGA
					GGCGGGCCAAGGTGG
Mimu-DRB*U080	1	110	163	yes	CAGCGGGTGCGGTACCTGGAGAGACACATCTACAACC
					GCGAGGAGATCGTGCGCTTCGACAGCGACGTGGGCGA
					GTTCCGGGCCGTGACGGAGCTGGGCCGGCCGAGCGCC
					GAGTACTTGAACCGCCAGCAGGACTACATGGAGCAGA
					CGCGGGCCTTGGTGG
Mimu-DRB*U081	1	583	163	yes	CAGCGGGTGCGGTTCCTGGAGAGACACATCTACAACC
					GCGAGGAGATCCTGCGCTTCGACAGCGACGTGGGCGA
					GTACCGGGCCGTGACGGAGCTGGGCCGGCCGGAAGCC
					GAGTACTGGAACCGCCAGCAGGACATCCTGGAGCAGA
					CGCGGGCGGAGGTGG
Mimu-DRB*U082	1	153	163	yes	CAGCGGGTGCGGTTCCTGGTGAGAGTCATCTACAACC
					GCGAGGAGTACGTGCGCTACGACAGCGACGTGGGCGA
					GTACCGGGCGGTGACGGAGCTGGGCCGGCCGGACGCC
					GAGTACTGGAACCGCCAGCAGGACCACCTGGAGCAGA
					GGCGGGCGGAGGTGG

The number of independent amplicons (*N* amp.) from which each sequence was retrieved, as well as the total number of reads (across runs and amplicons) for each sequence is given. The nucleotide sequence of new sequences retrieved from only one individual is shown in this table; such sequences were not submitted to public repository to ensure the storage of high quality sequences in such databases
